# Association Between Sleep Duration, Screen-Based Sedentary Time, and Weight Status Among Chinese Adolescents

**DOI:** 10.3390/healthcare13243237

**Published:** 2025-12-10

**Authors:** Masen Zhang, Jing Cui, Yuliang Sun

**Affiliations:** 1 Department of Physical Education, Northwestern Polytechnical University, Xi’an 710072, China; zhmasen@nwpu.edu.cn; 2 School of Nursing and Rehabilitation, Xi’an Fanyi University, Xi’an 710105, China; cj@xafy.edu.cn; 3 School of Physical Education, Shaanxi Normal University, Xi’an 710119, China

**Keywords:** overweight, obesity, adolescent, sleep duration, screen-based sedentary time

## Abstract

**Objectives**: The aim of this study was to examine the association between sleep duration, screen-based sedentary time, and overweight/obesity prevalence among Chinese adolescents. **Methods**: A cross-sectional analysis was conducted on 6174 adolescents in Shandong Province, China, covering general demographic characteristics, sleep time, screen-based sedentary time, physical activity, and other related variables. Height and weight measurements were obtained through on-site physical examinations, while other variable data were obtained through a questionnaire. The participants were categorized based on sleep time (<8 vs. ≥8 h/d) and screen-based sedentary time (<2 vs. ≥2 h/d). Logistic regression was employed to assess the independent and combined associations. **Results**: The overall prevalence of overweight in the adolescents was 27.1%. Adolescents with insufficient sleep (<8 h/d) had a significantly higher rate of overweight than those with adequate sleep (29.1% vs. 25.1%, *p* = 0.03). Similarly, those with high screen-based sedentary time (≥2 h/d) had a higher rate of overweight than those with low screen-based sedentary time (32.6% vs. 26.4%, *p* = 0.012). After adjustment for confounders, insufficient sleep and high screen time were independently associated with increased odds of being overweight. While no significant multiplicative interaction was found, the combination of insufficient sleep and high screen time presented the highest risk (OR = 1.552, 95% CI: 1.162–1.911). **Conclusions**: Both insufficient sleep duration and excessive screen-based sedentary time are independent risk factors for overweight/obesity among Chinese adolescents. A cumulative effect is suggested, as adolescents with both behaviors face the greatest risk. Public health interventions should concurrently promote adequate sleep and reduce screen-based sedentary time to combat adolescent overweight/obesity.

## 1. Introduction

Globally, the growing prevalence of adolescent overweight and obesity is a critical public health challenge, with significant implications for long-term health outcomes [1,2]. Data from the World Health Organization (WHO) reveal a marked escalation in pediatric overweight and obesity, with global prevalence among 5–19-year-olds climbing from 8% in 1990 to 20% in 2022, affecting more than 390 million individuals [3]. The trend is particularly concerning in China, where it is driven by rapid socioeconomic development and associated shifts in lifestyle behaviors [4]. A study on 210,168 Chinese students aged 6–17 showed that the overall prevalence of overweight rose to 38.60% in 2021, an increase of 6.52% in five years [5].

Adolescent obesity is a well-established predictor of numerous comorbidities, including osteoarthritis [6], diabetes [7], fatty liver disease [8], cancers [4], and impaired psychological health [9]. As the prevalence of overweight and obesity increases, these conditions are increasingly manifesting at earlier ages, underscoring the urgent need to identify modifiable risk factors [10]. Within this context, the 24 h movement paradigm—which encompasses physical activity, sedentary behavior, and sleep—has emerged as a crucial framework for understanding energy balance and weight status in young populations [10,11].

Among these behaviors, sleep duration and screen-based sedentary time are two factors that exhibit complex and potentially interconnected relationships with adiposity [12]. According to Dai, Chinese middle school students, on average, slept for 7.25 h per day, with more than 80% of adolescents getting less than the recommended 8 h of sleep per day in their age group. These issues need to be given attention [13,14]. A substantial body of evidence suggests that adolescents with short sleep duration have a higher incidence of obesity/overweight compared to those with recommended sleep [15,16], and insufficient sleep disrupts hormonal regulation, increasing the risk of obesity through mechanisms such as altered leptin and ghrelin levels and the expansions of two subcortical brain regions [15,17,18], and the incidence of obesity/overweight among Asians due to short sleep time showed significant results [19].

Concurrently, 21.5% of Chinese adolescents spend more than 2 h of screen time per day, and screen time was positively associated with weight status [20,21,22]. The school-based interventions that reduced television watching led to a decrease in body mass index (BMI) and other adiposity measures [23,24]. A study has shown that those who spend more time watching TV/videos have a 57.6% increase in overweight rates [25]. Excessive screen time not only displaces opportunities for physical activity but is also independently associated with increased energy intake, often through distracted snacking and exposure to food marketing [12]. For Chinese adolescents, who face intense academic pressures that often compromise sleep and encourage sedentary leisure activities, understanding the interplay between these behaviors is of paramount importance [26].

Despite existing research, significant knowledge gaps remain concerning the specific association between sleep time, screen time, and weight status with the unique context of Chinese adolescents. Many previous studies have examined these behaviors in isolation [15,17,18], failing to account for their intrinsic codependency within a finite 24 h day. Furthermore, there is a need for more robust investigations that consider how these factors may interact or have differential effects across various demographic subgroups, such as by gender or urban-rural residency.

Therefore, the purpose of this study was to examine the individual and combined associations of sleep duration and screen-based sedentary time with the weight status of Chinese adolescents. By addressing these research objectives, this study contributes evidence that can inform targeted public health interventions and policies designed to promote healthier lifestyles and combat the growing epidemic of adolescent obesity in China.

## 2. Materials and Methods

### 2.1. Participants

This study utilized data from the Chinese National Population Health Data Center for Database of Youth Health [27,28]. This study adopted a cross-sectional design, using the population proportionate sampling method to select middle schools based on geographical, demographic, and socio-economic level in Shandong Province, China. Those who completed the questionnaire information and physical examination were included in this study. Ultimately, a total of 6174 teenagers were selected in the 2020–2021 semester from 62 middle schools, of which 45.9% were boys (*n* = 2832, BMI = 21.9 ± 5.8 kg/m^2^, age = 13.1 ± 1.5 years) and 54.1% were girls (*n* = 3342, BMI = 20.4 ± 3.2 kg/m^2^, age = 12.9 ± 1.1 years).

Physical education teachers working in middle schools were recruited to participate in the investigation. They had experience in evaluating the physical fitness of adolescents and had implemented national student physical fitness testing projects [29]. To ensure standardization of testing and reduce testing errors, all teachers have completed two training sessions on testing procedures. Trained teachers used standardized guidelines to measure the height and weight, and guide them to answer questionnaires [27,30]. All participants signed informed consent forms before participating in the study, explicitly stated that all data was collected voluntarily, anonymously, and confidentially, kept on a password protected website, and was only accessed by direct researchers. The project was approved by the Ethics Committee of Shandong University (Approval No. 20180517).

### 2.2. Measurements

#### 2.2.1. Anthropometric Measurements

The adolescent participants’ height and weight were measured using a standardized digital electronic scale (HW-VB900, lEJIA, Hangzhou, China) (Table 1), and their body mass index (BMI) was calculated according to the formula weight/height squared (kg/m^2^). According to the Chinese national standard “Screening for Overweight and Obesity in School aged Children and Adolescents” (WS/T 586-2018) [31], the participants were categorized into two groups: those who are not overweight and those who are overweight.

#### 2.2.2. Questionnaire Survey

Data was collected using a self-administered questionnaire according to National Student Physical Fitness and Health Research handbook [27,28,32]. The key variables are described below.

Sleep duration: Adolescents’ sleep time was assessed by a question: “How long have you slept every night in the past 7 days?” Based on the hygiene requirements for daily study time of school students from National Health Commission of China and recommended amount of sleep for pediatric populations from the American Academy of Sleep Medicine”, middle school students should sleep for 8 h or more per day. Therefore, the participants were divided into two groups: <8 h/d and ≥8 h/d [33,34].

Screen-based sedentary time: Adolescents’ screen-based sedentary time was assessed by a question: “How much time have you spent on screens for non-educational purposes, including television, video games, mobile phones, computers, and other electronic devices in the past 7 days?” Following the guidelines for physical activity in the Chinese population and the public education’s guidelines from the American Academy of Pediatrics Committee, children and adolescents aged 6–17 should have a cumulative screen time of less than 2 h per day. Therefore, the participants were divided into two groups: <2 h/d and ≥2 h/d [35,36].

Covariates: Information on age, gender, place of residence (urban/rural), family economic status, and daily physical activity was also collected (Table 1). Adolescents’ daily physical activity was assessed by a question: “how many days did you engage in physical activities such as exercise, dancing, or vigorous physical activity in the past 7 days?”

### 2.3. Statistical Analysis

The analyses were conducted using SPSS 26.0 software (IBM, Chicago, IL, USA). Mean (M) and standard deviation (SD) values were used to present continuous variables (e.g., age), and independent sample *t*-tests were utilized for comparisons. Numbers and percentages (%) were used to present categorical variables, and Chi-square tests were utilized for comparisons. Logistic regression models were applied to evaluate the relationships between sleep duration, screen time, and overweight. Odds ratios (ORs) and their 95% confidence intervals (CIs) were calculated. Model 1 was the crude model. Model 2 was adjusted for gender, age, residence, and physical activity time. An interaction term (sleep time × screen time) was introduced into the logistic regression model to test for a multiplicative interaction effect. statistical significance was set at *p*-value < 0.05.

## 3. Results

### 3.1. The Prevalence of Overweight Among Adolescents

The final analytical sample consisted of 6174 adolescents (2832 boys and 3342 girls). The overall prevalence of overweight was 27.1%. As detailed in Table 2, significant disparities in the overweight rate were observed across different demographic groups. The prevalence was significantly higher in boys (36.4%) compared to girls (19.2%) (*p* < 0.001) and in urban adolescents (30.2%) compared to their rural counterparts (24.3%) (*p* < 0.001). No statistically significant differences were found concerning age (*p* = 0.150), family economic status (*p* = 0.587), or level of physical activity (*p* = 0.121).

Regarding the key exposure variables, 28.1% of adolescents reported sleeping less than 8 h/d, and 11.6% reported 2 h or more of screen-based sedentary time per day. The prevalence of overweight was significantly higher in adolescents with insufficient sleep (<8 h/d) compared to those with adequate sleep (*p* = 0.003). Similarly, a higher prevalence was found in adolescents with high screen time (≥2 h/d) compared to those with low screen time (*p* = 0.012).

### 3.2. Independent Associations of Sleep Duration and Screen-Based Sedentary Time with Overweight

The results of the logistic regression analysis are presented in Table 3. In the unadjusted model (Model 1), both short sleep duration and high screen-based sedentary time were significant risk factors for overweight. After adjusting for gender, age, place of residence, and physical activity time (Model 2), these associations were slightly strengthened and remained statistically significant. Adolescents sleeping less than 8 h/d had 1.256 times the odds of being overweight (95% CI: 1.085–1.535), and those with 2 h or more of screen-based sedentary time per day had 1.431 times the odds (95% CI: 1.103–1.758).

### 3.3. Combined Association of Sleep Duration and Screen-Based Sedentary Time with Overweight

The combined effect of sleep duration and screen-based sedentary time is shown in Table 4. The multiplicative interaction term between sleep time and screen time was not statistically significant. However, analysis of combined categories revealed a clear risk gradient. Using adolescents with adequate sleep (≥8 h/d) and low screen time (<2 h/d) as the reference group, those with adequate sleep but high screen time and those with insufficient sleep but low screen time did not show a statistically significant increase in risk. In contrast, adolescents exhibiting both risk factors—insufficient sleep and high screen time—faced the greatest risk, with a 55.2% increase in the likelihood of being overweight (95% CI: 1.162–1.911, *p* < 0.001).

## 4. Discussion

This cross-sectional study examined the associations between sleep duration and screen-based sedentary time with weight status among a sample of 6174 adolescents in Shandong Province, China. The key findings reveal that both insufficient sleep (<8 h/d) and excessive screen time (≥2 h/d) are significant independent risk factors for overweight/obesity. Furthermore, adolescents who exhibited both risk behaviors faced the highest risk, with 55.2% increased odds of being overweight compared to their peers with healthy sleep and screen habits.

Our findings are consistent with the growing body of evidence within the 24 h movement behavior framework. The independent association between short sleep duration and increased risk of overweight aligns with the results of previous studies. A study has shown that compared to the recommended sleep time group, participants in the short sleep time group had a 1.76 increase in the OR of overweight, a 1.69 increase in the OR of obesity, and a 1.49 increase in waist circumference [15]. A meta-analysis found that short sleep duration increased the risk of overweight or obesity in Chinese children and adolescents by 47% [16]. The reasons for overweight caused by insufficient sleep may be multifaceted. Sleep deprivation disrupts the balance of appetite-regulating hormones, such as by raising ghrelin levels (the hunger hormone) and lowering leptin levels (the satiety hormone) [37,38]. This hormonal shift can affect appetite and lead to increased energy intake. Furthermore, reduced sleep duration in children and adolescents may contribute to daytime sleepiness, fatigue, and impaired mood, which in turn leads to decreased physical activity and increased sedentary time, thereby elevating the risk of overweight and obesity [16]. For Chinese adolescents, who are often under significant academic pressure leading to chronic sleep deprivation [26], this pathway may be a particularly critical contributor to the obesity epidemic.

Similarly, our data confirm a positive association between high screen-based sedentary time (≥2 h/d)) and overweight risk, a relationship consistently documented in the literature [12,25]. A study has shown that the overweight rate among teenagers in Shanghai, China was 20.5%, and the overweight rate of those who spend more time watching TV/videos has increased by 57.6% [25]. A systematic review concluded that screen time durations exceeding 2 h/d were associated with higher levels of obesity and more depressive symptoms [39]. The mechanisms underlying this link are multifaceted and behavioral. First, adolescents who spend most of their leisure time on screens are unlikely to engage in sports activities. Screen time is an intrinsically sedentary behavior that directly displaces time that could be spent engaging in light or moderate-to-vigorous physical activity [40]. A study has shown that among children who spent less than 2 h a day on screen, only 13% did not participate in physical activity, a number increased sharply as screen time increased, and was strongly correlated with BMI [41]. Second, it is frequently accompanied by mindless snacking on energy-dense, nutrient-poor foods. An extra hour of screen time a day is associated with approximately 3.3 extra servings of sugary beverages and 7.1 extra servings of unhealthy snacks per week [42]. Furthermore, exposure to targeted advertising for unhealthy foods and beverages [42], screen use can strongly shape food preferences and promote poor dietary choices. Screen time is related to low vegetable intake and high intake of sweets and soft drinks, and ultimately leads to obesity [43]. Our results affirm that screen time is a significant and modifiable risk factor that demands targeted attention in public health strategies aimed at curbing adolescent obesity. However, it is critical to emphasize that the type of screen use (educational and recreational), not merely its duration, is the key consideration. While our study only included recreational screen time, and demonstrated a positive correlation with adolescent overweight, other studies showed no significant association between educational screen time and weight status [25]. Therefore, it is necessary to consider the content and differentiate the use of recreational and educational screens when minimizing screen-based activities.

A particularly important finding of this study is the combined effect of short sleep and high screen time for overweight. Although no statistically significant multiplicative interaction was detected, the additive effect is stark and clinically meaningful. The group with both risk factors had the highest odds of overweight (OR = 1.552). This suggests a cumulative risk pattern in which these two habits may compound each other’s negative effects. Using screens, especially when exposed to blue light at night, can inhibit the production of melatonin, delay the onset of sleep, disrupt sleep structure, and exacerbate pre-existing sleep deprivation [44]. A study has shown that excessive screen-based sedentary time was significantly correlated with poor sleep quality and higher daytime sleepiness [45]. Conversely, the fatigue and reduced cognitive control resulting from poor sleep may diminish an individual’s capacity for physical activity and increase the tendency for sedentary escapism, often manifested as prolonged screen time for passive entertainment [16]. A study has found that when children get less sleep, they appear to spend more time engaged with screens, and use screens more as a percentage of their day when tired [46]. This establishes a vicious cycle: poor sleep promotes more screen time [47], and more screen time further degrades sleep [45], a feedback loop that collectively reduces energy expenditure, disrupts circadian-regulated hormones like leptin and ghrelin, and ultimately promotes a positive energy balance and weight gain [12,16,48]. Schools and parents should be encouraged to create environments that support early sleep, restrict the use of digital devices [49], and help adolescents establish healthy lifestyles during the critical period of habit formation through early intervention, break the vicious cycle of “screen excess/sleep deprivation leading to obesity”, and lay the foundation for their lifelong health.

This research has a few limitations. Primarily, its cross-sectional nature prevents determining causal links. Longitudinal studies are necessary to verify the direction of these relationships. Second, other potential variables, such as dietary patterns, pubertal stage, or psychological factors, which may potentially influence the estimated associations, were not adequately controlled for. Third, although data were collected across multiple schools, the clustered sampling structure was not incorporated into the analysis, which may result in underestimated standard errors and inflated statistical significance. Additionally, data on sleep and screen time were self-reported and may be influenced by recall or social desirability biases. Future research would benefit from objective measurements collected with tools such as accelerometers and screen-time-tracking applications. Despite these limitations, this study has some advantages, including a relatively large sample size, the use of objective height and weight measurements, and the investigation of both the independent and combined effects of two key modifiable behaviors.

## 5. Conclusions

In conclusion, both insufficient sleep and excessive screen-based sedentary time are significant risk factors for overweight in Chinese adolescents. The risk is most pronounced for individuals who exhibit both behaviors simultaneously. These findings have crucial consequences for public health strategies and clinical practice. Interventions aimed at preventing and managing adolescent obesity should adopt an integrated approach that addresses multiple health behaviors within the 24 h cycle. Specifically, recommending that health promotion efforts should stress the need for enough sleep and less recreational screen-based sedentary time. Schools and parents should be encouraged to create environments that support earlier bedtimes and limit access to digital devices, especially in the evening. Future interventions should target these modifiable lifestyle factors to effectively curb the rising trend of adolescent overweight/obesity in China. Nonetheless, given the cross-sectional nature of this study, causal inferences cannot be drawn, and longitudinal studies should further verify the direction of these relationships.

## Figures and Tables

**Table 1:** The measurement of this study and variable assignment.

Variable	Measurement	Assignment
Height	Stand straight against the stadiometer, look directly ahead, while the height (to 0.1 cm) is measured.	\
Weight	Stand straight against the stadiometer, look directly ahead, while the weight (to 0.1 kg) is measured.	\
Gender	What is your gender?	0 = boy, 1 = girl
Sleep duration	How long have you slept every night in the past 7 days?	0 = <8 h/d, 1 = ≥8 h/d
Screen time	How much time have you spent on screens for non-educational purposes, including television, video games, mobile phones, computers, and other electronic devices in the past 7 days?	0 = <2 h/d, 1 = ≥2 h/d
Residence	Is your household registration location in urban or rural area?	0 = urban, 1 = rural
Family economic status	How is your family’s economic status?	0 = poor, 1 = average, 2 = good
Physical activity	how many days did you engage in physical activities such as exercise, dancing, or vigorous physical activity in the past 7 days?	0 = 0 d, 1 = 1 d, 2 = 2–3 d, 3 = 4–5 d, 5 = 6–7 d

**Table 2:** Comparison of overweight rate of adolescents in different groups.

Group	Non-Overweight	Overweight	*t* or *X*^2^	*p*
Age ^a^	13.0 ± 1.3	12.8 ± 1.5	2.43	0.150
Gender ^b^				
Boys	1802 (63.6)	1030 (36.4)	114.29	<0.001
Girls	2700 (80.8)	642 (19.2)		
Residence ^b^				
Urban	2022 (69.8)	876 (30.2)	13.69	<0.001
Rural	2480 (75.7)	796 (24.3)		
Family economic status ^b^				
Poor	776 (72.5)	294 (27.5)	1.06	0.587
Average	3350 (73.3)	1220 (26.7)		
Good	376 (70.4)	158 (29.6)		
Physical activity ^b^				
None	2372 (74.5)	814 (25.5)	7.35	0.121
1 d	806 (73.5)	290 (26.5)		
2–3 d	870 (68.9)	392 (31.1)		
4–5 d	274 (71.4)	110 (28.6)		
6–7 d	180 (73.2)	66 (26.8)		
Sleep time ^b^				
<8 h/d	1228 (70.9)	504 (29.1)	4.74	0.003
≥8 h/d	2630 (74.9)	882 (25.1)		
Screen time ^b^				
<2 h/d	4018 (73.6)	1438 (26.4)	6.24	0.012
≥2 h/d	484 (67.4)	234 (32.6)		

Note: h/d means hours a day; ^a^ Mean ± Standard deviation; ^b^ Number (Percentage).

**Table 3:** Independent associations of sleep time and screen time with overweight among adolescents.

	Group	Overweight (%)	Model 1		Model 2	
			OR (95% CI)	*p*	OR (95% CI)	*p*
Sleep time	≥8 h/d	882 (25.1)	1.00		1.00	
	<8 h/d	504 (29.1)	1.224 (1.020, 1.468)	0.030	1.256 (1.085, 1.535)	0.021
Screen time	<2 h/d	1438 (26.4)	1.00		1.00	
	≥2 h/d	234 (32.6)	1.351 (1.066, 1.711)	0.013	1.431 (1.103, 1.758)	0.008

**Table 4:** Combined association of sleep duration, screen time, and overweight among adolescents.

Sleep Time	Screen Time	OR	95% CI	*p*
Sleep time × screen time		1.249	(0.951, 1.639)	0.109
≥8 h/d	<2 h/d	1.00		
≥8 h/d	≥2 h/d	1.186	(0.961, 1.262)	0.086
<8 h/d	<2 h/d	1.070	(0.899, 1.321)	0.321
<8 h/d	≥2 h/d	1.552	(1.162, 1.911)	<0.001

## Data Availability

Detailed data supporting the results can be obtained through the following website: https://dyh.sdu.edu.cn/index.htm (accessed on 1 August 2025).
